# Unraveling bacterial fingerprints of city subways from microbiome 16S gene profiles

**DOI:** 10.1186/s13062-018-0215-8

**Published:** 2018-05-22

**Authors:** Alejandro R. Walker, Tyler L. Grimes, Somnath Datta, Susmita Datta

**Affiliations:** Department of Biostatistics, University of Florida, 2004 Mowry Rd, Gainesville, FL 32610 USA

**Keywords:** Microbiome, Bacterial 16S gene, Classifier, PCA, Network analysis, Machine learning

## Abstract

**Background:**

Microbial communities can be location specific, and the abundance of species within locations can influence our ability to determine whether a sample belongs to one city or another. As part of the 2017 CAMDA MetaSUB Inter-City Challenge, next generation sequencing (NGS) data was generated from swipe samples collected from subway stations in Boston, New York City hereafter New York, and Sacramento. DNA was extracted and Illumina sequenced. Sequencing data was provided for all cities as part of 2017 CAMDA contest challenge dataset.

**Results:**

Principal component analysis (PCA) showed clear clustering of the samples for the three cities, with a substantial proportion of the variance explained by the first three components. We ran two different classifiers and results were robust for error rate (< 6%) and accuracy (> 95%). The analysis of variance (ANOVA) demonstrated that overall, bacterial composition across the three cities is significantly different. A similar conclusion was reached using a novel bootstrap based test using diversity indices. Last but not least, a co-abundance association network analyses for the taxonomic levels “order”, “family”, and “genus” found different patterns of bacterial networks for the three cities.

**Conclusions:**

Bacterial fingerprint can be useful to predict sample provenance. In this work prediction of provenance reported with over 95% accuracy. Association based network analysis, emphasized similarities between the closest cities sharing common bacterial composition. ANOVA showed different patterns of bacterial amongst cities, and these findings strongly suggest that bacterial signature across multiple cities are different. This work advocates a data analysis pipeline which could be followed in order to get biological insight from this data. However, the biological conclusions from this analysis is just an early indication out of a pilot microbiome data provided to us through CAMDA 2017 challenge and will be subject to change as we get more complete data sets in the near future. This microbiome data can have potential applications in forensics, ecology, and other sciences.

**Reviewers:**

This article was reviewed by Klas Udekwu, Alexandra Graf, and Rafal Mostowy.

## Background

The advent of NGS technologies has experienced a tremendous effect on –omics applications. The reduction of costs since its introduction [1] has accelerated the use of this technology on metagenomics experiments [2, 3]. Phylogenetic survey analyses based on 16S gene diversity have been fundamental on identification of bacterial varieties [4–6]. This sequencing revolution, in conjunction with high performance computing, and recently developed computing tools has had a vast impact on new 16S gene studies [5, 7]. The use of WGS data on microbiome experiments has been widely reported and has multiple advantages when compared with 16S amplicon data [8].

**Fig. 1:**
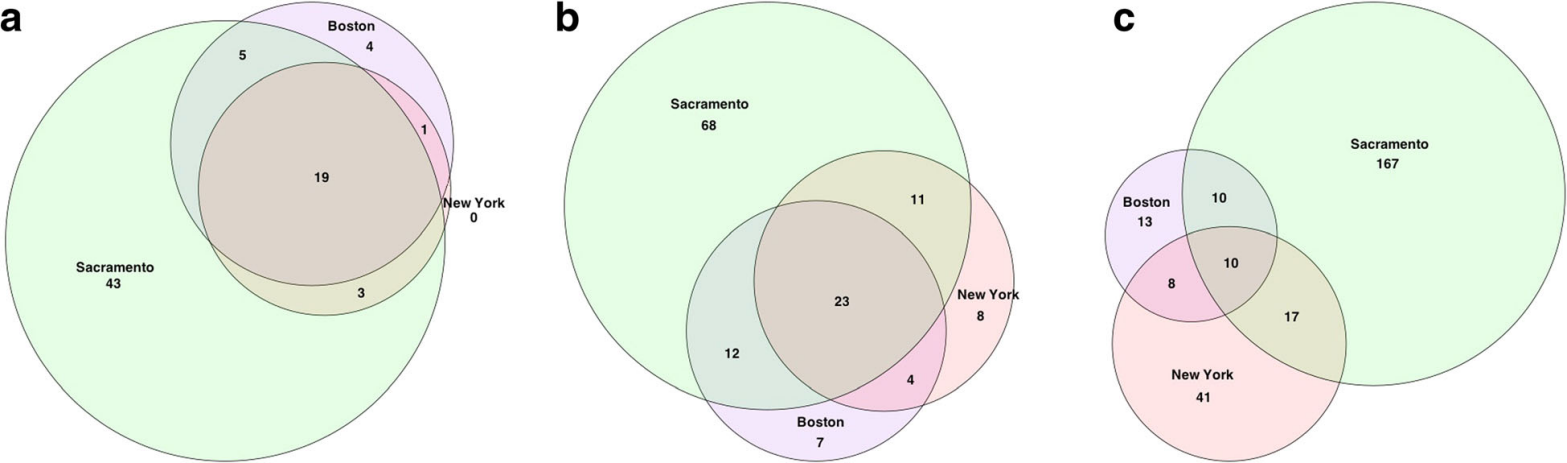
Area-proportional Venn Diagrams of discovered entries across all three taxonomic ranks. **a**), **b**), and **c**) represent the counts for taxonomic ranks “order”, “family”, and “genus”, respectively. Three cities intersection represents the count of common variables used for most of the analyses in this work. Total count for each city represents the effective number of species (S)

In this work, we focus on the MetaSUB Challenge dataset as part of the 2017 CAMDA competition. MetaSUB International Consortium aims to create longitudinal metagenomic map of mass-transit systems, and other public spaces around the world. They partnered with CAMDA for an early release of microbiome data of Boston, New York, and Sacramento for the massive data analysis challenge. Swab samples collected from subway stations in these three cities, were Illumina-sequenced at variable depths, and provided for further analyses in compressed FASTQ format. The data set consisted of 141, 1572, and 18 samples from Boston, New York, and Sacramento, respectively (Table 1). Subsequent bioinformatics processing was conducted in the “HiPerGator” high performance cluster at the University of Florida. Sequence data files were uncompressed, quality filtered, and open-reference operational taxonomic units (OTUs) were picked using QIIME pipeline [9]. After quality control, the effective number of samples included in this work was 134 in Boston, 777 in New York, and 18 in Sacramento (Table 1). OTUs were aggregated as counts and normalized for three taxonomic ranks. The selected ranks were “order”, “family”, and “genus”, based on the number of common levels across all three cities (see Fig. 1). A summary of the common levels for each taxonomic rank is also presented in Table 1.

**Table 1:** Sample count for city and effective samples analyzed and resulting number of common entries for each of the selected taxonomic ranks included in this work

City	Effective samples	Total samples	Order	Family	Genus
New York	777	1572	19	23	10
Boston	134	141			
Sacramento	18	18

Our motivation is to unravel the bacterial fingerprints of all these three different cities (similarities and differences) using only common bacterial signatures within three taxonomic ranks. In particular, we consider four different statistical analyses; each is conducted across cities using a common taxonomic rank, and the analysis is repeated for each rank. The analyses include PCA, sample provenance prediction using classification techniques, differential abundance of bacteria across cities using ANOVA, and network analysis based on statistical association of bacterial signatures.

## Results

### Principal component analysis

**Fig. 2:**
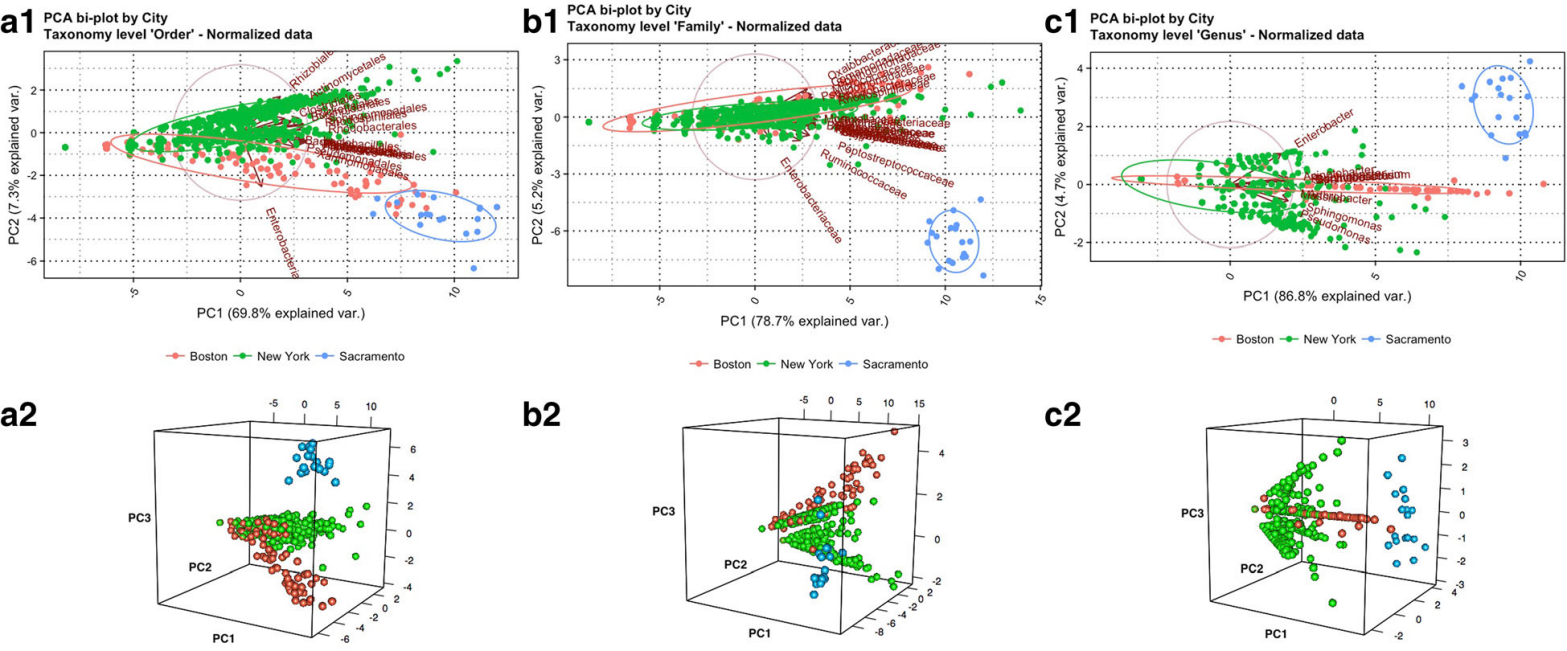
PCA bi-plots of principal components 1, and 2 are presented in **a1**, **b1**, and **c1** for taxonomic ranks “order”, “family”, and “genus”, respectively. Three-dimensional plots of first three components are presented in **a2**, **b2**, and **c2** for taxonomic ranks “order”, “family”, and “genus”, respectively. Colors are: orange for Boston, green for New York, and blue for Sacramento

First we describe the results of our PCA conducted on these samples. Table 2 presents a summary of the variability explained by the first three components. As seen in this summary, the total amount of variance explained by the first 3 principal components was consistently greater than 80% for all taxonomic ranks. Plots of principal components are presented in Fig. 2, sorted by taxonomic ranks with “order” on the left and “genus” on the right. The top row illustrates bi-plots of components 1 and 2 with a remarkable clustering of the samples from the three cities. As seen in all three plots (A1, B1, and C1), the majority of variables with each taxonomic rank were highly correlated with the first principal component (being nearly parallel to the corresponding axis). On the other hand, as seen in plot A1, the “order” *enterobacteriales* showed a higher correlation with the second principal component. This might highlight a low importance of this “order” for Boston, and New York. This was also concordant in plots B1, and C1 for “family” *enterobacteriaceae*, and “genus” *enterobacter*, respectively. Second row in Fig. 2 presents three-dimensional (3D) plots of first 3 components (A2, B2, and C2). The clustering of the cities is even more clear-cut from these 3D-plots. These plots, along with the bi-plots, also support the premise that Boston, and New York both have similar bacterial patterns compared with Sacramento.

**Table 2:** Total amount of variance explained by principal components 1-3 for all three taxonomic tanks (“order”, “family”, and “genus”)

Rank	PC1	PC2	PC3	Total
order	69.8%	7.3%	6.7%	83.8%
family	78.7%	5.2%	4.7%	88.6%
genus	86.8%	4.7%	3.1%	94.6%

### Classification analysis

Class prediction of city of origin was conducted using in two different approaches. First, prediction of sample provenance was carried out using the Random Forest [10] classifier (RF). This is a well-regarded classifier for its superior theoretical and practical performances, and is robust to over fitting. The model was fitted for each taxonomic rank. The overall classification error rates were 3.01, 3.12, and 6.77% for “order”, “family”, and “genus” respectively; note that RF calculates these rates internally by using the out-of-bag error of samples. Results for each city are presented in Table 3. The error rate for “genus” was somewhat elevated compared to the other two, perhaps as a consequence of having less features (10) compared to the other two (19, and 23). The classification error for New York samples was particularly low, probably because of the large amount of sequencing data available for this city. Sacramento also showed low classification errors even though the data set had only 18 samples for this city. However, as shown even by our PCA, these samples had a distinctive bacterial signature compared to the other two making them easier to identify by a classifier such as RF. Overall, the Boston samples were the hardest to distinguish possibly due to their similarity with New York samples. Perhaps a larger representative sample from Boston would produce a better classifier.

**Table 3:** Random forest classification error of city across all taxonomic ranks “order”, “family”, and “genus”

City	Order	Family	Genus
	Number of predictors		
	19	23	10
	Classification error		
Boston	11%	18%	34%
New York	1%	1%	2%
Sacramento	6%	0%	11%

**Fig. 3:**
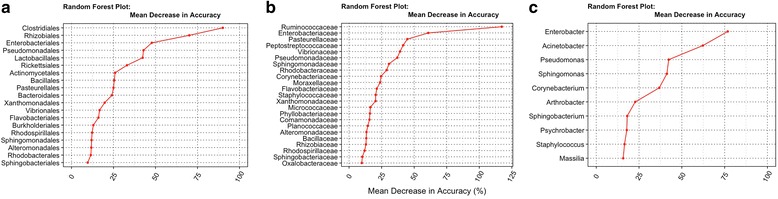
Variable importance for the Random forest classifier, as determined by the mean decrease in accuracy. **a**), **b**), and **c**) are importance plots for taxonomic ranks “order”, “family”, and “genus” respectively

The importance of each predictor can be measured based on the mean decrease in accuracy when the predictor is removed from the model; these results are presented in Fig. 3. In plot A, the top three “orders”, namely *clostridiales*, *rhizobiales*, and *enterobacteriales* are the most effective in predicting a city. Interestingly, in plot B, the top “families” belong to the same top “orders” from plot A. On the other hand, the top “genera” in plot C did not correspond to those in plots A and B.

**Fig. 4:**
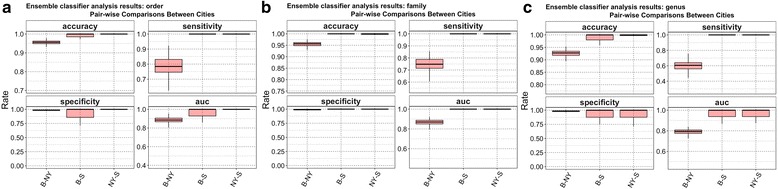
Ensemble results, in terms of Accuracy, Sensitivity, Specificity, and AUC for each taxonomic rank. **a**), **b**), and **c**) correspond to taxonomic rank “order”, “family”, and “genus” respectively. Each individual plot shows pairwise classification results for comparisons of Boston – New York, Boston – Sacramento, and New York – Sacramento

The second approach we implemented was an Ensemble [11] classifier (EC), which is restricted to binary predictions. Results are presented (see, Fig. 4) in terms of classification accuracy, sensitivity, specificity, and area under the curve (AUC). Ensemble results showed that prediction accuracy, and sensitivity for Boston-Sacramento (B-S), and New York-Sacramento (NY-S) pairs were consistently over 98% for all taxonomic ranks. It is interesting to note that the overall accuracy for the three-city classification system was only slightly worse as shown in the previous paragraph for RF results. Accuracy, and sensitivity results for Boston-New York (B-NY) pair were smaller - 92, and 60%, respectively, both at taxonomic rank “genus”. Specificity results were the best for B-NY and worst for B-S for all ranks. AUC was generally greater than 95% across all three ranks, although at taxonomic rank “genus” appeared to have a large variation.

### Differential abundance analysis

Analysis of variance for taxonomic rank “order” revealed that bacterial abundance is highly significantly different for most of the common levels across the three cities. Table 4 shows minimum, averaged, and maximum *p*-values, and counts for each “order” across the three cities, reported for the corresponding Tukey group after 5000 replications. It can also be inferred from Table 4 that city means for the first four orders were all significantly different across city (group a-b-c), with a small percentage of the samples (< 5%) corresponding to Tukey’s group a-a-b. Additionally the top 11 order means were significantly different in all the replications and were in a large number of them counted as a-b-c (> 30%) and in some others as a-a-b. The analysis also found a few features that were significantly different only in a small number of replications, proving the effectiveness of the balanced ANOVA. These orders were *sphingomonadales*, and *rhodospirillales*, with 324 and 649 significant cases respectively.

**Table 4:** ANOVA results for taxonomic rank “order”. Tukey’s multiple comparison test results after 5000 replications significant *p*-values (*α* = 0.01) were averaged and counted for Tukey’s groups (Boston-New York-Sacramento). In general terms, when comparing two cities if letters (‘a’, ‘b’ and ‘c’) are all the same, we conclude that the means are not significantly different. If the letters are different we conclude city means are significantly different in terms of bacterial abundances. As for example, “order” *enterobacteriales*, shows minimum, average and maximum *p*-value out of 5000 replications, and 4967 times out of 5000 replications the three city means were found to be significantly different ‘a’-‘b’-‘c‘; 30 times Boston and New York mean bacterial abundances remain the same but Sacramento is different (‘a’-‘a’-‘b’) and only in 3 cases Boston, and Sacramento are the same but New-York (‘a’-‘b’-‘a’) is different deemed by Tukey’s multiple comparison test. Taxonomic rank names (“order”) are presented in the same order for all groups (‘a’-‘b’-‘c’, ‘a’-‘a’-‘b’, ‘a’-‘b’-‘b’, ‘a’-‘b’-‘a’)

Order	*p*-value [min, mean, max]	Count	Tukey’s group
Enterobacteriales	[8.45E-17, 3.08E-8, 1.46E-5]	4967	a-b-c
Clostridiales	[1.75E-30, 2.42E-19, 3.13E-16]	4802	
Rhizobiales	[5.18E-16, 3.37E-6, 3.25E-4]	4693	
Pseudomonadales	[5.37E-15, 6.63E-6, 1.27E-3]	4432	
Xanthomonadales	[1.14E-16, 2.01E-11, 3.38E-9]	3134	
Flavobacteriales	[1.24E-20, 1.46E-14, 7.18E-12]	1939	
Vibrionales	[4.51E-19, 2.3E-13, 1.89E-11]	1939	
Sphingobacteriales	[8.56E-16, 1.41E-11, 1.7E-9]	1889	
Bacillales	[5.03E-19, 9.78E-14, 1.99E-11]	1562	
Alteromonadales	[8.15E-23, 1.22E-16, 4.13E-14]	1478	
Bacteroidales	[1.09E-26, 5.25E-18, 4.08E-15]	1474	
Rhodobacterales	[9.85E-8, 2.91E-4, 4.53E-3]	1332	
Pasteurellales	[2.74E-18, 1.04E-12, 2.15E-10]	1187	
Rickettsiales	[4.03E-9, 7.4E-4, 5.75E-3]	683	
Lactobacillales	[4.54E-27, 2.6E-17, 5.45E-15]	667	
Actinomycetales	[1.9E-7, 8.94E-4, 4.56E-3]	157	
Burkholderiales	[6.92E-8, 1.26E-3, 5.4E-3]	95	
Sphingomonadales	[1.76E-8, 9.08E-4, 3.62E-3]	43	
Rhodospirillales	[2.34E-6, 6.66E-4, 2.78E-3]	24	
Enterobacteriales	[6.64E-11, 6.3E-7, 8.58E-6]	30	a-a-b
Clostridiales	[2.6E-24, 6.04E-19, 3.07E-17]	198	
Rhizobiales	[3.29E-8, 8.12E-5, 1.8E-3]	288	
Pseudomonadales	[2.58E-8, 9.45E-5, 2.22E-3]	187	
Xanthomonadales	[6.43E-17, 4.36E-11, 4.84E-9]	1866	
Flavobacteriales	[4.06E-21, 4.38E-14, 7.69E-12]	3061	
Vibrionales	[4.48E-20, 5.02E-13, 7.33E-11]	3061	
Sphingobacteriales	[1.01E-16, 4.25E-11, 3.17E-8]	3111	
Bacillales	[6.69E-20, 1.19E-13, 9.96E-11]	3438	
Alteromonadales	[1.87E-24, 3.04E-16, 7.47E-14]	3522	
Bacteroidales	[7.59E-28, 1.91E-17, 2.23E-14]	3526	
Rhodobacterales	[6.23E-8, 9.45E-4, 9.91E-3]	3249	
Pasteurellales	[3.69E-19, 2.08E-12, 3.97E-10]	3813	
Rickettsiales	[3.36E-7, 4.13E-3, 9.96E-3]	1234	
Lactobacillales	[1.14E-25, 1.13E-16, 6.48E-14]	4333	
Actinomycetales	[1.01E-5, 4.01E-3, 9.95E-3]	1028	
Burkholderiales	[2.04E-5, 4.94E-3, 9.74E-3]	110	
Sphingomonadales			
Rhodospirillales	[4.49E-4, 5.1E-3, 9.91E-3]	57	
Enterobacteriales			a-b-b
Clostridiales			
Rhizobiales	[4.35E-9, 4.86E-5, 4.36E-4]	19	
Pseudomonadales			
Xanthomonadales			
Flavobacteriales			
Vibrionales			
Sphingobacteriales			
Bacillales			
Alteromonadales			
Bacteroidales			
Rhodobacterales			
Pasteurellales			
Rickettsiales	[3.84E-7, 2.83E-3, 9.99E-3]	568	
Lactobacillales			
Actinomycetales	[1.18E-5, 2.72E-3, 9.32E-3]	61	
Burkholderiales	[6.45E-9, 2.92E-3, 10E-3]	1584	
Sphingomonadales	[1.63E-5, 3.33E-3, 9.99E-3]	209	
Rhodospirillales	[5.42E-3, 7.22E-3, 10E-3]	5	
Enterobacteriales	[6.46E-11, 6.52E-9, 1.94E-8]	3	a-b-a
Clostridiales			
Rhizobiales			
Pseudomonadales	[1.38E-12, 5.01E-6, 4.48E-4]	381	
Xanthomonadales			
Flavobacteriales			
Vibrionales			
Sphingobacteriales			
Bacillales			
Alteromonadales			
Bacteroidales			
Rhodobacterales	[9.6E-8, 1.02E-3, 9.81E-3]	320	
Pasteurellales			
Rickettsiales	[3.52E-4, 4.95E-3, 8.88E-3]	27	
Lactobacillales			
Actinomycetales	[1.01E-5, 3.83E-3, 9.88E-3]	261	
Burkholderiales	[6.81E-3, 6.81E-3, 6.81E-3]	1	
Sphingomonadales	[7.24E-5, 4.92E-3, 9.75E-3]	62	
Rhodospirillales	[1.43E-6, 4.15E-3, 9.97E-3]	559	

Effective number of species (S) found in all cities across the three taxonomic ranks, is shown as proportional-area Venn diagram in Fig. 1. The plot shows greater diversity in Sacramento compared with both Boston, and New York for all taxonomic ranks also the diversity increases, as taxonomic rank moves from “order” to “genus”. Mean species diversity (*α*_*t*_) [12, 13] were calculated for all taxonomic ranks across cities (see eq. (5)) for two values for the weight modifier “q” (0.5, and 2.0). Using bootstrap based test [14] results (see Table 5) showed that mean species diversity (q = 0.5) was significantly different (*α* = 0.05) for taxonomic ranks “order”, and “family”. For “genus”, test for Mean species diversity between the three cities was borderline significant. Results for the second weight modifier (q = 2) showed that mean species diversity, across all taxonomic ranks, was not significant in our bootstrap analysis. These opposing results, for values of the weight modifier, can be interpret as an over-inflated weight of low abundance species in the mean species diversity when q = 0.5, hence the number of time when the sum of squares deviated from the real value was low. Conversely when q = 2 high abundance species have a larger effect in the mean species diversity calculations.

**Table 5:** Bootstrap results (replications = 2000) for mean species diversity across all taxonomic ranks. Table shows *p*-values for two values of weight modifier (0.5, and 2)

q	Order	Family	Genus
0.5	0.004	0.006	0.084
2	0.930	1.000	0.999

### Network analysis

**Fig. 5:**
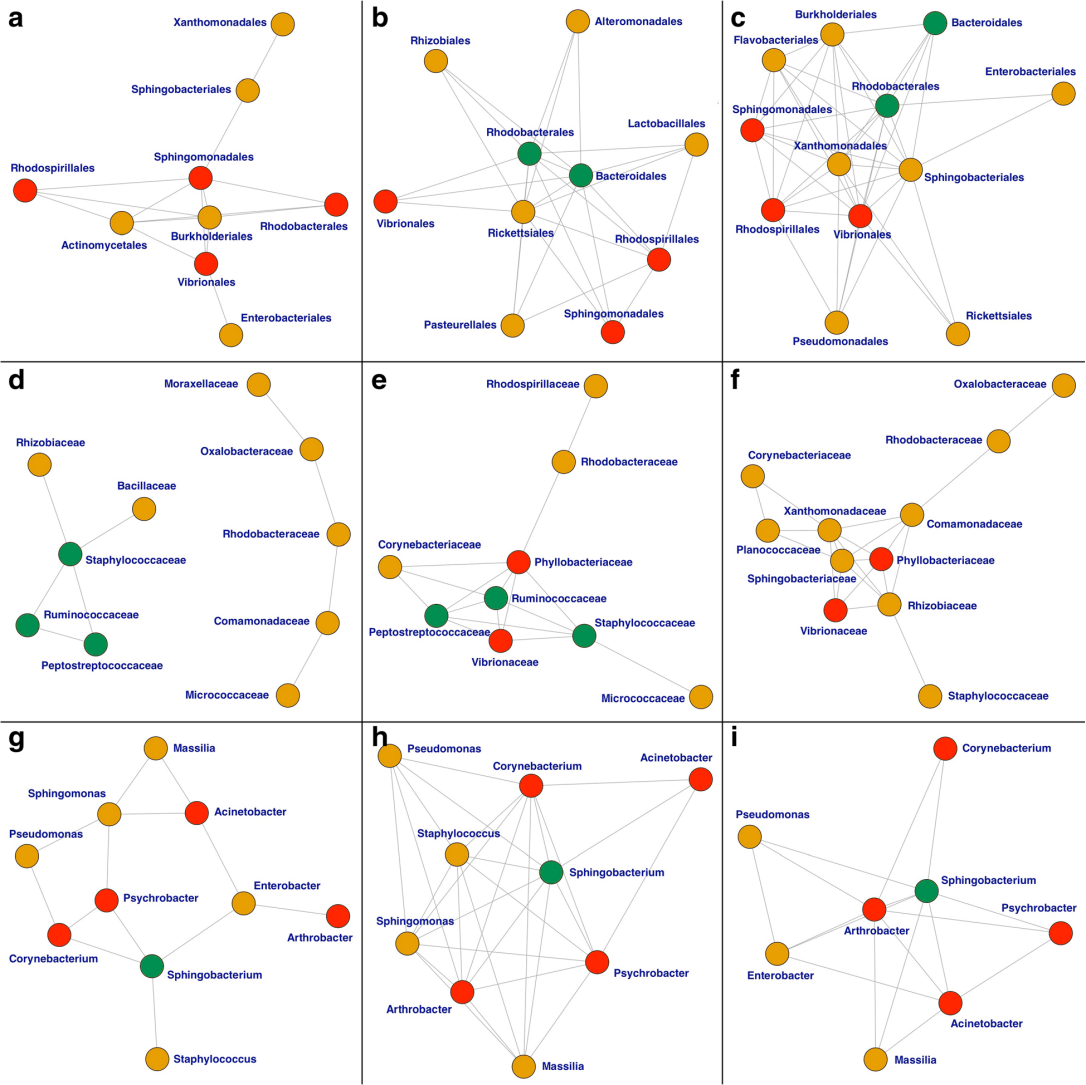
Abundance association networks for the three cities based on bacterial fingerprints using common OTUs. Left column corresponds to networks from Sacramento, CA; middle column are networks from New York, NY; and right column from Boston, MA. Top row has networks for the taxonomic rank “order”, middle row is for the taxonomic rank “family”, and bottom row is for “genus”

Networks presented in Fig. 5 are purposely placed geographically, west on the left, and east on the right. The first row depicts the networks for each city for taxonomic rank “order.” Plots in the top row show “orders” *rhodobacteriales*, and *bacteroidales* (green) as highly connected nodes for east cities, which belong to higher taxonomic rank “class” *alphaproteobacteria*, and *bacteroidia*, respectively. Nodes in red are those “orders” found across all cities, all belonging to “classes” *alphaproteobacteria* and *gammaproteobacteria*. Networks for taxonomic ranks “family” in the second row, show an interesting change across cities, with central nodes in red that are common between Boston and New York and nodes in green that are common between New York and Sacramento. The last row shows networks for taxonomic ranks “genus”. In all cities we can identify a sub-structure with a hub node in green corresponding to the “genus” *sphingobacterium*. This central node shares four highly connected nodes (in red) for the east-coast cities but lose complexity for the city of Sacramento as the number of connections for each node drops considerably compared with the other two cities. In general we have found that cities of Boston and New York have more complex networks for all taxonomic ranks when compared with networks from Sacramento.

## Discussion and conclusion

It has been well established that WGS metagenomics can fail to detect rare species since DNA is not sequenced with enough depth as a result of its rarity [15, 16]. Nevertheless, this was not an issue for the development of this work since our main objective was to determine the common bacterial signature of the three cities in the form of normalized counts of taxonomic ranks and use this data to predict the source of origin of a specific sample. We present a set of tools complementing, rather than competing with one another, in characterizing the differential signatures in terms common bacteria. Overall the different analytical components of this work, collectively, conveyed the following consistent message: The bacterial signatures of common OTUs, are city specific in terms of normalized counts for the three taxonomic ranks.

PCA findings showed a large proportion of the variability (> 80%) is accounted for by the first three principal components for the three taxonomic ranks. Prediction of provenance based on bacterial fingerprints was also highly effective (classification error < 6%, and accuracy > 90%) for all classifiers tested, although the classifiers performed better for ranks “order”, and “family” as a result of having more common predictors (19, and 23 respectively). ANOVA showed that the bacterial signature is city specific with specific patterns of differentiation. While ANOVA showed differential bacterial patterns across cities, the effective number of species diversity showed that Sacramento had the largest number of species. This can be the result of warmer climate condition of Sacramento that promotes bacterial growth and ecological diversity compared with the colder climates of Boston, and New York, but we note that the result may be biased by the effect of uneven “wet lab” protocols for DNA extraction and sequencing, and very unequal city sample sizes, although we tried to deal with the later issue by subsampling. Finally, network analysis showed that each city has a different overall bacterial network structure. A careful review of nodes from Boston, and New York revealed common subnetwork-structures sharing similar bacterial patterns, which is believed to be result of geographic proximity, and common ecological niche for northeast coastal cities contrasting with a southwestern city in California. Network analyses for future datasets with a more balanced design, and more standardized DNA extraction and sequencing protocols, might lead to interesting ecological perspectives regarding species that live in mutualism or symbiosis, and others that show patterns of competition.

The results presented in this work, all support the fact that it is possible to capture the bacterial signal from samples collected in three cities using OTUs counts from common bacteria; nevertheless it is definitely possible that the quality of the results and conclusions could be greatly improved if a review of the experimental-design lead to a more balanced number of samples for each city, combined with objective-specific protocols for DNA extraction and sequencing of the samples, which should ensure a more uniform sequencing depth and quality, specially across cities. As a closing remark the authors emphasize that these analyses were conducted on preliminary data and results are a valuable source to plan future experiments and analyses.

## Methods

For the 2017 meeting, CAMDA has partnered with the MetaSUB (Metagenomics & Metadesign of Subways & Urban Biomes) International Consortium (http://metasub.org/), which has provided microbiome data from three cities across the United States as part of the MetaSUB Inter-City Challenge.

Illumina next generation sequencing data was generated from swab DNA samples taken on subway stations from Boston, New York, and Sacramento. Data was provided in the form of FASTQ files for each sample, plus a supplementary dataset with information regarding swab places, sequencing technology, DNA extraction, and amplification, samples names, etc. A quality control of the reads was conducted to improve taxonomical classification with QIIME. The raw OTUs generated with QIIME, were aggregated for each sample to generate a matrix of OTUs counts for the three cities. The subsequent statistical analyses were conducted on the basis of common OTUs, finding additional patterns in the relative abundance that was not as obvious as the presence of city-specific OTUs. Other aspects of bio-diversity beyond what is apparent from Fig. 1 (such that Sacramento samples exhibited the most biodiversity) were not investigated further.

### Sequencing data description

Boston sequencing data consisted of a total of 141 samples ranging from 1 Mbp to 11 Gbp single read Illumina data. The majority of the samples (117 Amplicon samples) were target sequenced after PCR amplification. Additionally, the rest of the samples (34) were whole genome shotgun (WGS) sequenced. Moreover, a small fraction of the amplicon samples did not effectively contribute to OTU counts, and hence they were removed from the analyses. Ultimately a total of 134 samples were included in further downstream analyses.

All 1572 New York samples were WGS, ranging from 0 Mbp to 19 Gbp of Illumina-sequence data. After quality control a subset of 777 samples effectively yielded OTU counts and were included in all subsequent analyses.

In the city of Sacramento, six locations were sampled three times each on different surfaces for a total of 18 WGS sequenced samples ranging from 2.8 to 3.4 Gbp. All the samples contained enough sequencing data after quality control to positively contribute to OTU counts, therefore all 18 samples were included in all the analyses.

### Bioinformatics and data processing

Sequencing data from each city was uncompressed and quality filtered to ensure improved OTU picking. Filtering FASTQ files was done with FASTX-Toolkit [17] at variable Phred quality scores ranging from 35 to 39 with a variable minimum percent of bases that must satisfy the chosen quality averaged score ranging from 40 to 80. This filtering scheme was designed for the purpose of effectively reducing the size of the large FASTQ files without compromising the open-reference OTU picking and for keeping the computational burden in check. This strategy not only accomplished the later goal but also removed the low quality FASTQ files which were unusable for detecting any 16S gene signal; The reduced sample sizes and their distributions according to the taxonomic ranks are provided in Table 1. This quality control yielded sequencing data in the order of a few Mbp up to 5 Gbp as a maximum. It is noteworthy that we processed amplicon FASTQ files with the same approach. In the study we merged WGS (only the 16S region) and Amplicon data in a combined fashion in order to have enough sample size. However, in order to establish the similarity of data distribution for the two platforms, we implemented a Kolmogorov-Smirnov test of the equality of the distributions comparing the data from both the platforms for each one of the features or levels found for the three taxonomic ranks. The null hypothesis states that the empirical distribution of the normalized counts from the WGS data is not significantly different from the empirical distribution of the normalized counts for the Amplicon data. Results confirmed that the data from both platforms are similar enough to be used together for further downstream analyses. No significant *p*-values were found in the Kolmogorov-Smirnov test (p-value_min_ = 0.2387 and p-value_max_ = 0.9945).

Filtered FASTQ files were converted to FASTA files with a “bash” script in order to standardize the description line for each sequence making it acceptable for QIIME pipeline. This step was required since we faced some incompatibility between FASTA files automatically generated by open-source converters. OTUs picking was conducted with QIIME in open-reference mode. This strategy was preferred since our purpose is to effectively detect the 16S gene region from as many bacterial species as possible. QIIME pipeline was run in three steps.

1 $$ \mathrm{pick}\_\mathrm{open}\_\mathrm{reference}\_\mathrm{otus}.\mathrm{py}-\mathrm{o}./\mathrm{otus}-\mathrm{i}./\mathrm{sample}.\mathrm{fa}-\mathrm{p}../\mathrm{parameters}.\mathrm{txt}-\mathrm{f}-\mathrm{a}-\mathrm{O}\ 12 $$

2 $$ \mathrm{biom}\ \mathrm{convert}-\mathrm{i}./\mathrm{otu}\mathrm{s}/\mathrm{otu}\_\mathrm{table}.\mathrm{biom}-\mathrm{o}./\mathrm{otu}\mathrm{s}/\mathrm{from}\_\mathrm{biom}.\mathrm{txt}--\mathrm{to}-\mathrm{tsv} $$

3 $$ \mathrm{assign}\_\mathrm{taxonomy}.\mathrm{py}-\mathrm{i}./\mathrm{pynast}\_\mathrm{aligned}\_\mathrm{seqs}/\mathrm{aligned}.\mathrm{fasta}-\mathrm{m}\ \mathrm{rdp} $$

The first step was the open reference OTU picking (1). The second was to convert the binary biom table into a text format output (2). The final step corresponds to assigning taxonomy values to all OTUs within the output table (3). OTU output counts were later aggregated at three taxonomic ranks as input data for further statistical analyzes. In other words, those OTUs that by mapping score are different, but correspond to the same taxonomic rank are added and labeled as the corresponding taxonomic rank they belong.

The chosen taxonomic ranks were “order”, “family”, and “genus”. Figure 1 presents a summary of aggregated OTUs for all the ranks. The selection of ranks was determined by the count of common levels within each threshold. The raw data for each taxonomic rank was then normalized to log counts per million for each city before combining them in a single dataset. The normalization was done based on Law et al. work [18] given in Formula (4). The OTU proportions (transformed) were calculated for each sample by

4 $$ {y}_{gi}={\mathit{\log}}_2\left(\frac{r_{gi}+0.5}{N{R}_i+1}{10}^6\right), $$

where *r*_*gi*_ is the *g*^*th*^ OTU count for sample *i*, *N* is the number of OTU categories, and ${R}_i=\frac{1}{N}\sum \limits_{g=1}^G{r}_{gi}$ is the mean number of mapped reads for *i*^*th*^ sample. This normalization scheme guarantees that the counts are bounded away from zero by 0.5 to make the logarithm meaningful and to reduce the variability of log-cpm for lowly expressed OTUs. Additionally, the library size was offset by 1. Together these guarantees that the ratio is strictly less than 1 and greater than zero.

### Statistical analysis

The proceeding statistical analysis was conducted in multiple stages in R [19]. The first was a PCA, which showed that the normalized counts for all taxonomic ranks carry strong enough signals to group the cities of origin. The second was to build a statistical classifier, which can produce a well-defined rule (e.g., a machine) to predict the city of origin from the rank profiles of a sample. To this end, we used two well-regarded classifiers, all within the R environment, and compared the findings. In a third stage we conducted a differential abundance analysis using ANOVA and a novel bootstrap based test using the alpha diversity indices. The final stage was to implement a visual inspection of the co-abundance networks in order to assess how the bacterial abundances vary jointly across the cities.

### Principal components analysis (PCA)

Unsupervised learning of normalized count data through principal component analysis was conducted on a taxonomic rank basis for “order”, “family”, and “genus”. The analysis was entirely conducted in R based on correlations structure. Eigenvalues were extracted to calculate the variability in the dataset accounted by each component. Two-dimensional PCA bi-plots, and three-dimensional plots of the first three components were generated for each taxonomic rank and color-coded by city to better visualize patterns amongst samples from each location (Fig. 2).

### Classification analysis

Accurately predicting the origin of a sample only based on common bacterial metagenomics is another objective of this work. We used two well-regarded classifiers to address this problem: random forest [10], and the adaptive optimal ensemble classifier [11].

The random forest (RF) classifier has improved classification accuracy as result of choosing vectors randomly and independently with a positive impact on the growth of each tree within the ensemble. This algorithm is robust to over-fitting (see theorem 1.2 in [10]), computationally efficient, and calculates estimates for class-specific mean decrease in accuracy, and internal error. RF was implemented with 10 variables or levels within each taxonomic rank, randomly chosen at each split, with 1000 trees. Results are provided in Table 3.

**Fig. 6:**
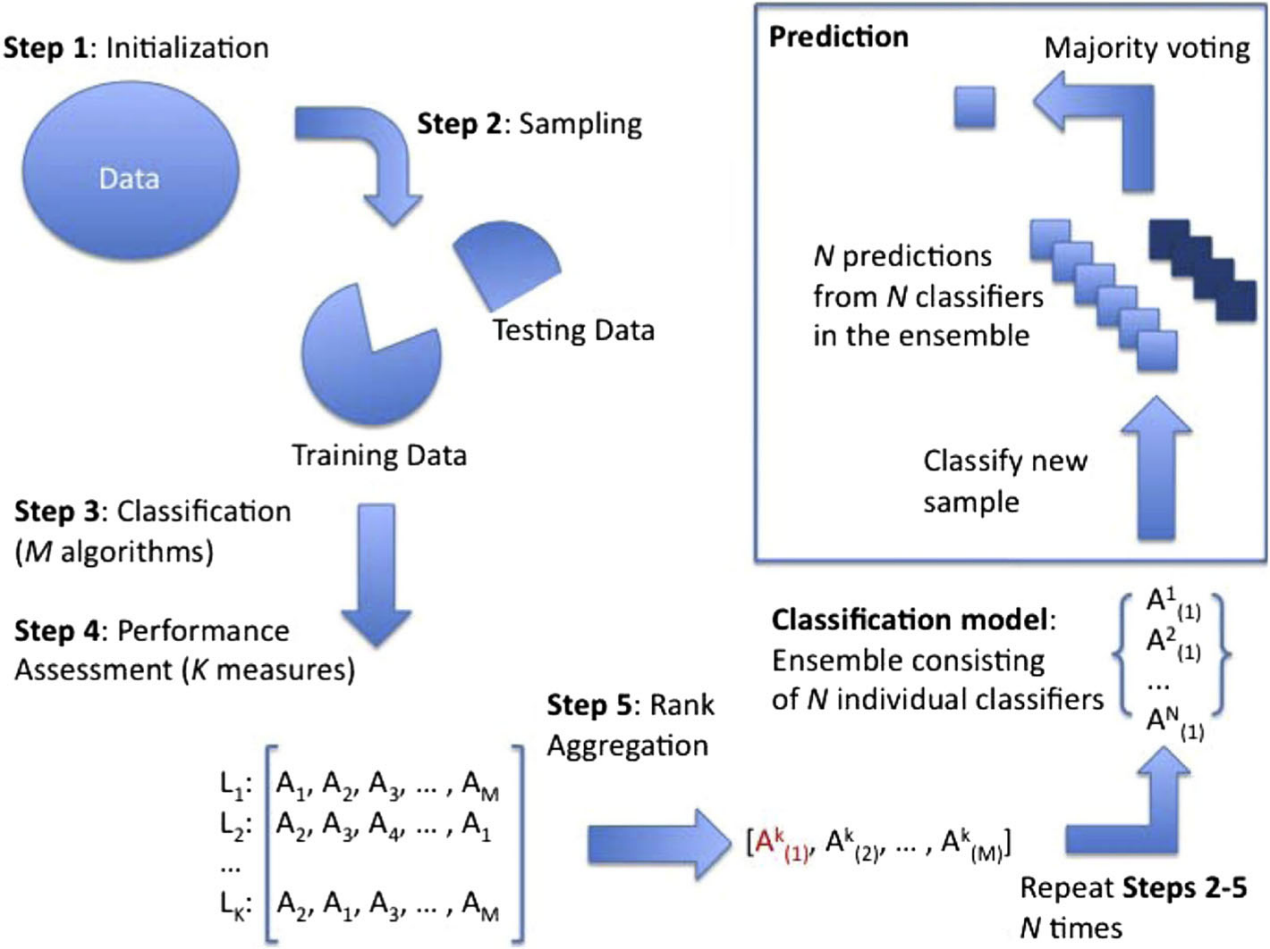
Workflow of the ensemble classifier (reproduced from Datta et al. [11])

Next we describe the implementation of the ensemble classifier (EC). As the name suggests, it is based on a number of individual (or component) classifiers. Figure 6 depicts the workflow of the ensemble classifier. Steps 1 and 2 prepare the dataset for training, and testing, followed by steps 3 to 5, which are classification, performance assessment, and rank aggregation. Ultimately, step 6 corresponds to prediction, and voting. EC is, however, restricted to binary classifications, so we separated the dataset into three pairwise sets. For each pairwise comparison, the analysis was conducted on a 2-fold training-test cross validation run for 100 iterations. The results are reported in Fig. 4.

### Differential abundance analysis

One-way analysis of variance of common taxonomic rank across cities was performed for each common level within taxonomic rank “order”. Due to the unbalanced nature of the dataset (refer to Table 1), we randomly subsampled cities of Boston and New York in subsets of 18 samples each, which correspond to the number of samples of the third city of Sacramento. On this balanced data sets we run the ANOVA analysis and repeat this for a total of 5000 replicates. The null hypothesis (H_0_) is that mean log-transformed normalized bacterial counts are equal across cities, and the alternate hypothesis (H_a_) is that at least one of the means is not equal to the others. We have controlled the FDR at 1% level for the multiple hypotheses correction. Additionally, we assess which of the three cities are different for each bacteria for the taxonomic rank “order” entries, by implementing Tukey’s multiple comparison test [20]. We reported the number of times each “order” was significantly different, the minimum, average, and maximum *p*-value, and also the pattern of the differences in terms of three letters (‘a’, ‘b’ and ‘c’) in Table 4.

Last but not the least, we investigated whether there were significant differences for the mean species diversity [12, 13] of order “q” calculated as follow,

5 $$ {\alpha}_t=\frac{1}{\sqrt[q-1]{\sum \limits_{j=1}^N\sum \limits_{i=1}^S{p}_{ij}{p}_{i\mid j}^{q-1}}}, $$

where *p*_*ij*_ is the proportional abundance of species *i* within sampling unit *j*, *p*_*i* ∣ *j*_ is the conditional proportions of species *i* given sampling unit *j*, *S* is the number of entries found in each taxonomic rank (species richness), and “*q*” is the weight modifier. In (5), *α*_*t*_ is conditional to the sampling unit (city) and values were calculated for two weight modifiers (0.5, and 2.0). As “*q*” takes the value 0.5, the abundance is intermediate between the harmonic mean (*q*=0) and the geometric mean as *q* approaches 1. The function represents the arithmetic mean when *q*=2. A bootstrap [14] approach was implemented on the basis of the dataset containing all species discovered (raw counts), to determine how consistent the mean species diversity was across cities. A total of *N* = 2000 bootstrap samples were generated by randomly changing the city vector on the data set, keeping the same number of samples, for each city as in the original dataset. A sum of squares across cities was calculated and tested as statistic (6),

6 $$ \theta ={\left({\alpha}_B-\overline{\alpha}\right)}^2+{\left({\alpha}_{NY}-\overline{\alpha}\right)}^2+{\left({\alpha}_S-\overline{\alpha}\right)}^2, $$

where *α*_*B*_, *α*_*NY*_, and *α*_*S*_ are alpha diversities within cities and $\overline{\alpha}$ is the mean alpha diversity. This statistics *θ* was also calculated for all bootstrap samples as *θ*^∗^ (n=1, …, 2000) and *p*-value was calculated as follow,

7 $$ p- value=\frac{1}{N}\sum \limits_{n=1}^{2000}I\left({\theta}_n^{\ast }>\theta \right) $$

Results are provided in Table 5.

### Network analysis

Network construction is often used in the context of gene-gene, gene-protein or protein-protein association/interaction networks [21]. However, one may use the correlation of the transformed and normalized OTU counts to construct a “co-abundance” network. In this study, we applied Pihur et al.’s strategy [22] in conjunction with the *dna* R package [23] to identify connectivity of bacterial fingerprints across three different cities for each taxonomic rank and visually identify the similarity and differential structure of them. Graphical networks were generated with the *network.modules* function (*dna*), which calls the *plot* function from R package *igraph* [24]. A matrix of Pearson’s correlations was generated for common entries, across taxonomic ranks for each city. Network plots were constructed connecting the edges with absolute correlation values greater than a threshold, which is specific for each network. Thresholds for cities at each taxonomic rank were chosen on a case-by-case basis in order to keep a similar number of nodes at each city.

## Reviewers’ comments

### Reviewer’s report 1: Klas Udekwu

Reviewer’s comments: The article ‘Unraveling bacterial fingerprints of city subways from microbiome 16S gene profiles’ details the comparative analysis of 16S derived bacterial signatures carried out using a statistiscal analyses (ANOVA) and PCAs as well as network analysis of association. The study is well-designed and describes adequately for the most part. The authors describe a city specific microbiome fingerprint from their analysis ov variance between the three chosen cities. While some issues still require attention, the results of the analysis as presented are clear and the methods used are adequate. Some of the methods although insufficiently described, are novel in such application and on the whole this represents a significant The tense used throughout the article however, should be maintained and the table and figure formats required.

1. Firstly, the numbering of the lines is off and disturbing, several of the statistical tables can be combined for simplicity and the figures need some higher resolution.
  Author’s response:
*First, we want to thank the reviewer for his comments and suggestions. In the current submission we have removed the line numbers and only used the numbers added by the submission manager. Regarding the tables, we carefully reviewed them and decided not to merge them because the rows/columns in these tables have little overlap. Figures are created in high resolution now.*
2. MAJOR concerns: here is little or no discourse regarding the size of the three datasets, the quality and discussion regarding disparities therein.
  Author’s response:
  *To address this issue we have now created multiple balanced data sets by randomly subsampling from the original New York data. We show that the analysis results of these balanced data are large consistent across various replicates* (Table 4)*. See the methods, results and conclusions of the revised paper for details.*
3. Concluding from three datasets of different weights, quality and provenance that city specificity is discernable without qualifying the caveats adequately is inadvisable.
  Author’s response:
*We do recognize and correct for the presence of multiple sources of biases related to these data sets that were provided for the CAMDA challenge. We have included comments regarding these aspects in multiple places in the manuscript; see, for example, in the last but one line of the Conclusions section. However, our goal was to provide a possible data analysis pipeline for such data and to demonstrate that microbiome data collected from the city subways do possess classification abilities even after adjustments for various artificial sources of biases.*
4. I suggest the authors change the wording slightly to reflect the necessity for more datasets being included in the study. The use of ‘expression’ to term bacterial abundance in several places in the text reflects transcriptomics and NOT metagenomics. Please correct where appropriate. The weight modifier set at different levels leads to completely different outcomes. The authors should discuss this. Figures require higher resolution even for submission as it is impossible to discern some of the text in the Figs 2, 3, 4.
  Author’s response:
*We have addressed this within the revised manuscript.*
5. The last line of page 7 is indicative of the hurdles one leaps in order to conclude as the authors do; ‘the signature is city specific (only) in terms of NORMALIZED counts of OTUs for three taxonomic ranlks.
  Author’s response:
*That is correct. In particular, we wanted to ensure that the signatures are robust and not due to technical differences between the samples from different cities.*
6. I would appreciate a discussion regarding normalization approaches they considered and how they settle on this.
  Author’s response:
*Please look at Equation (* *4* *) of the manuscript. This normalization scheme, has been used by many in the microbiome community. We have additionally included some discussions in the manuscript (right after Eq. (* *4* *)) to provide the rational behind this normalization.*
7. A subset of randomized samples of equal number and even representation irrespective of diversity, analyzed in the same way would have given the reader more confidence in the conclusions.
  Author’s response:
  *As the results of ANOVA gets affected by unequal sample size we have modified the ANOVA analysis considering random subsamples of equal size (18, the same as the sample size of Sacramento) from the New York and Boston samples and conducted the ANOVA analysis. The reported results are then based over all the subsamples and demonstrate a large degree of consistency* (see, Table 4)*. Nevertheless, we recognize that a more balanced experiment with similar “wet-lab” protocols would give stronger conclusion. However, we were restricted to the size of the data available for CAMDA 2017. We include some discussion regarding this in the Discussion and Conclusion sections.*

### Reviewer’s report 2: Alexandra Graf

Reviewer’s comments: The study tries to find city specific metagenome fingerprints. It uses several classical statistics and machine learning methods to analyze the data from three different cities (New York, Sacramento and Boston) provided by the CAMDA challenge. Without cell count the abundances measured in metagenome datasets are only relative abundances. Any kind of comparison based on the differences in abundance between samples, are questionable and will probably not result in a microbial profile indicative of the cities real species composition. Especially with such varying sequencing depths as seen between the 3 cities as well as their differing experimental approach (Amplicon, WGS). Differences that are seen between the cities could stem from all kinds of technical biases during sampling, DNA extraction and sequencing.

1. The study uses QIIME to predict OTUs, using only the 16S rRNA data. This enables the authors to make use of all samples (16S and WGS) but introduces a bias, since the 16S rRNA sequence extractions from whole genome data behave differently than Amplicon sequenced data.
  Author’s response:
*We want to thank Dr. Graf for her valuable comment. Ideally, one would only use one platform for data collection. It is to be noted that we neither had any control over the quality and quantity of the data nor the experimental design. We did not want to discard the Amplicon samples because that would have led to a substantially reduced sample size for Boston. However, Dr. Graf’s point is well taken. To that end, we have tested that the distributions of the normalized data from the two platforms are similar using a Kolmogorov-Smirnov test. See the “Bioinformatics and Data processing” section under “Methods” (pages 11-12) for the details.*
2. Furthermore, it is not discussed which 16S rRNA region was used in the Amplicon sample preparation. But it is known that different regions show a taxonomically biased result.
  Author’s response:
*Unfortunately, since the samples were provided as part of the 2017 CAMDA MetaSUB Challenge, there is no further information regarding this issue, and after reviewing the counts we obtained from Amplicon samples, and how comparable they were with those from WGS samples, we decided to move on with the analyses.*
3. The sample size differs considerably between the cities (134 Boston, 777 NY, 18 Sacramento after QC filter) which influences the statistical analysis considerably. The amount of sequence data differs considerably between the samples, which has an influence on the taxonomic content of the samples (< 1 Mbp to 19 Gbp), as will the non-microbial proportion of the data, which also differs considerably between the samples.
  Author’s response:
*The point is well taken. However, as stated earlier we did not have any control over the experimental design as the data were provided from the CAMDA 2017 challenge. We have modified the ANOVA analysis in order to address the unbalanced nature of the dataset by considering random subsamples of equal size (18, the same as the sample size of Sacramento) from the New York and Boston samples and conducted the ANOVA analysis multiple times and reported finding that were consistently supported.*
4. The authors also talk about differential expression (Page 6, Line 9 and Table 4), which obviously cannot be inferred from genomic DNA data. And as stated before also different abundances of species between different samples cannot be inferred from the analyzed data.
  Author’s response:
*We have changed the writing in the revised manuscript.*

### Reviewer’s report 3: Rafal Mostowy

Reviewer’s comments: The article by Alejandro Walker and colleagues takes on a challenge of using a computational approach to analyse microbiome data from three locations (NY, Boston, Sacramento), and distinguish the location from microbial composition alone based on 16 s rRna sequencing. The authors propose several different approaches to tackle the problem, including principal component analysis, two machine learning methods (Random Forest and Ensemble), differential abundance analysis and network analysis. They find compositional differences between the three locations using all approaches, and thus conclude that microbiome data can have potential applications in forensics and other sciences. As a non-expert in microbiome research, I’m writing this review from a perspective of a computational biologist. I find the problem very interesting and the diverse set of approaches used by the authors valuable. It is always reassuring to observe similar patterns using very different methods (like PCA and regression for example). Thus, the conclusion that bacterial composition differs with location is quite well supported in this study. So clearly, the paper makes a valuable contribution to our understanding of whether we can guess a location based on a microbiome sample from this location.

1. I can’t help but feel that it is a shame that the MS does not go a step or two further and give recommendations regarding potential pros and cons of different approaches. In other words, the paper’s punchline is that microbiome compositions indeed differ by location, and that is probably a prior expectation of almost everyone reading this paper.
  Author’s response:
*We thank you for your overall positive assessment. However, we tend to disagree somewhat with your view of the “punchline”. As you expressed so correctly, we also understand that as a prior expectation. However, we provided a set of tools complementing, rather than competing with one another, in characterizing these differential signatures. We have clarified this point in the revised paper – see the conclusion and discussion section.*
2. The interesting thing about this paper is that the differences are captured by such a variety of methods, but the authors do not really provide the reader with any understanding about what aspects of microbial compositions (or differences between them) these methods capture. One suggestion would be to include a complementary benchmarking effort to compare how well those approaches do in detecting real differences (or particular aspects of compositional differences). Such data could be generated in silico, and robustness of different approaches with respect to detecting changes in microbial compositions could be analysed.
  Author’s response:
*This paper grew out of the CAMDA 2017 MetaSUB Challenge, and we investigated an aspect of the data set provided to participants. We agree that with a more comprehensive dataset (or simulation studies) and comparative statistical analyses using that would be of considerable value. However, that is beyond the scope of this paper.*
3. Furthermore, with regards to how useful different methods are in finding compositional differences, I’m yet to be convinced about the value of the network analysis in this context. It is certainly a nice idea to use abundance-similarity networks, but I would expect either a more thorough analysis of the resulting networks using a more formal statistical approach, or a biological interpretation of the results. Otherwise, I’m not sure about the point of using such networks. It would be good if the authors addressed this in the MS.
  Author’s response:
*We have reviewed the manuscript regarding this comment, and we are convinced that network analysis provides a joint representation of all the common OTUs together in terms of abundances and at least visually observe whether the topology of the networks in three different cities are the same or not. This can also give a broad insight on how bacterial populations are interacting, and how their ecological niche on occasion overlaps depending on geographic proximity.*
4. Finally, I think that the explanation of the classification approach could be a little better. In particular, I don’t quite understand what the authors used as a predictor. Was it a mere presence of the OTU unit, its frequency or something else, and why? Please explain.
  Author’s response:
  *We have modified the manuscript with regard to the way we generated the data for the classifiers. This can be easily understood by looking at* Figure 1*, where the intersections for the three cities show 19, 23 and 10 species for “order”, “family”, and “genus”. The improved description of how the dataset was generated, which can be found in page 12 and 13. This gives a clear idea on how the counts were aggregated, how and why only three taxonomic ranks were chosen for further analyses. As a closing statement we can say that the strength of the manuscript is that even with the availability of this partial dataset disclosed out of the CAMDA 2017 challenge data initiative and considering a subset of the common “bugs” we can reach to some interesting scientific conclusions which can ultimately be validated further with the forthcoming bigger datasets of CAMDA 2018. I hope the revised manuscript provides a more comprehensive understanding of the predictors.*

## Abbreviations

ANOVA: Analysis of variance
AUC: Area under the curve
EC: Ensemble classifier
NGS: Next generation sequencing
OTU: Operational taxonomic unit
PCA: Principal component analysis
RF: Random forest classifier
WGS: Whole genome sequencing

## References

[1] Metzker ML (2010). Applications of next-generation sequencing sequencing technologies - the next generation. Nat Rev Genet.

[2] Thomas T, Gilbert J, Meyer F (2012). Metagenomics - a guide from sampling to data analysis. Microbial informatics and experimentation.

[3] Simon C, Daniel R (2011). Metagenomic analyses: past and future trends. Appl Environ Microb.

[4] Clifford RJ, Milillo M, Prestwood J, Quintero R, Zurawski DV, Kwak YI, Waterman PE, Lesho EP, Mc Gann P. Detection of bacterial 16S rRNA and identification of four clinically important Bacteria by real-time PCR. PLoS One. 2012;7(11):1–6. 10.1371/journal.pone.0048558 PMC3490953 23139793

[5] Caporaso JG, Lauber CL, Walters WA, Berg-Lyons D, Lozupone CA, Turnbaugh PJ, Fierer N, Knight R (2011). Global patterns of 16S rRNA diversity at a depth of millions of sequences per sample. Proc Natl Acad Sci U S A.

[6] Kuczynski J, Stombaugh J, Walters WA, Gonzalez A, Caporaso JG, Knight R (2011). Using QIIME to analyze 16S rRNA gene sequences from microbial communities. Current protocols in bioinformatics.

[7] Tringe SG, Hugenholtz P (2008). A renaissance for the pioneering 16S rRNA gene. Curr Opin Microbiol.

[8] Ranjan R, Rani A, Metwally A, McGee HS, Perkins DL (2016). Analysis of the microbiome: advantages of whole genome shotgun versus 16S amplicon sequencing. Biochem Biophys Res Commun.

[9] Caporaso JG, Kuczynski J, Stombaugh J, Bittinger K, Bushman FD, Costello EK, Fierer N, Pena AG, Goodrich JK, Gordon JI (2010). QIIME allows analysis of high-throughput community sequencing data. Nat Methods.

[10] Breiman L (2001). Random forests. Mach Learn.

[11] Datta S, Pihur V, Datta S (2010). An adaptive optimal ensemble classifier via bagging and rank aggregation with applications to high dimensional data. BMC bioinformatics.

[12] Tuomisto H (2010). A diversity of beta diversities: straightening up a concept gone awry. Part 1. Defining beta diversity as a function of alpha and gamma diversity. Ecography.

[13] Whittaker RH (1972). Evolution and measurement of species diversity. Taxon.

[14] Efron B, Tibshirani R (1993). An introduction to the bootstrap.

[15] Shah N, Tang H, Doak TG, Ye Y (2011). Comparing bacterial communities inferred from 16S rRNA gene sequencing and shotgun metagenomics. Pacific symposium on Biocomputing Pacific symposium on Biocomputing.

[16] Kalyuzhnaya MG, Lapidus A, Ivanova N, Copeland AC, McHardy AC, Szeto E, Salamov A, Grigoriev IV, Suciu D, Levine SR (2008). High-resolution metagenomics targets specific functional types in complex microbial communities. Nat Biotechnol.

[17] Patel RK, Jain M (2012). NGS QC toolkit: a toolkit for quality control of next generation sequencing data. PLoS One.

[18] Law CW, Chen YS, Shi W, Smyth GK. Voom: precision weights unlock linear model analysis tools for RNA-seq read counts. Genome Biol. 2014;15(2):1–17. 10.1186/gb-2014-15-2-r29 PMC4053721 24485249

[19] Team RDC (2010). R: a language and environment for statistical computing.

[20] Tukey JW (1949). Comparing individual means in the analysis of variance. Biometrics.

[21] Gill R, Datta S, Datta S. A statistical framework for differential network analysis from microarray data. BMC bioinformatics. 2010;11:1–10. 10.1186/1471-2105-11-95 PMC2838870 20170493

[22] Pihur V, Datta S, Datta S (2008). Reconstruction of genetic association networks from microarray data: a partial least squares approach. Bioinformatics.

[23] Gill R, Datta S, Datta S (2014). dna: an R package for differential network analysis. Bioinformation.

[24] Csardi G, Nepusz T (2006). The igraph software package for complex network research. InterJournal.

